# The effects of anxiety and dual-task on upper limb motor control of chronic stroke survivors

**DOI:** 10.1038/s41598-020-71845-7

**Published:** 2020-09-15

**Authors:** Mahnaz Hejazi-Shirmard, Laleh Lajevardi, Mehdi Rassafiani, Ghorban Taghizadeh

**Affiliations:** 1 Department of Occupational Therapy, School of Rehabilitation Sciences, Iran University of Medical Sciences, Tehran, Iran; 2 Rehabilitation Research Center, Department of Occupational Therapy, School of Rehabilitation Sciences, Iran University of Medical Sciences, Tehran, Iran; 3 Occupational Therapy Department, Faculty of Allied Health Sciences, Kuwait University, Kuwait City, Kuwait; 4 Neurorehabilitaion Research Center, The University of Social Welfare and Rehabilitation Sciences, Tehran, Iran

**Keywords:** Cerebrovascular disorders, Neuroscience

## Abstract

This study was designed to investigate the effects of anxiety and dual-task on reach and grasp motor control in chronic stroke survivors compared with age- and sex-matched healthy subjects (HC). Reach and grasp kinematic data of 68 participants (high-anxiety stroke (HA-stroke), n = 17; low-anxiety stroke (LA-stroke), n = 17; low-anxiety HC, n = 17; and high-anxiety HC, n = 17) were recorded under single- and dual-task conditions. Inefficient reach and grasp of stroke participants, especially HA-stroke were found compared with the control groups under single- and dual-task conditions as evidenced by longer movement time (MT), lower and earlier peak velocity (PV) as well as delayed and smaller hand opening. The effects of dual-task on reach and grasp kinematic measures were similar between HCs and stroke participants (i.e., increased MT, decreased PV that occurred earlier, and delayed and decreased hand opening), with greater effect in stroke groups than HCs, and in HA-stroke group than LA-stroke group. The results indicate that performing a well-learned upper limb movement with concurrent cognitive task leads to decreased efficiency of motor control in chronic stroke survivors compared with HCs. HA-stroke participants were more adversely affected by challenging dual-task conditions, underlying importance of assessing anxiety and designing effective interventions for it in chronic stroke survivors.

## Introduction

Approximately 60% of stroke survivors suffer from permanent upper limb dysfunctions despite receiving rehabilitation^1^. Stroke-induced motor impairments (e.g., muscle weakness, spasticity, and impaired coordination), sensory deficits (proprioceptive and/or tactile sensory loss) and perceptual-cognitive dysfunctions (e.g., attentional problems and visuospatial impairments), as well as secondary physiological adaptations (e.g., contractures, and muscle atrophy) can directly affect skilled/well-learned upper limb movements such as reach and grasp^2^. Slowed and segmented movement, dysmetria, inadequate aperture and impairments of hand preshaping have been reported as common problems, involved in clumsy function or disuse of the upper limb following stroke^2–5^. Reach and grasp, a fundamental part of object manipulation, requires the integration of sensory, motor and cognitive information^6,7^ and frequently performed with a concurrent cognitive task (e.g., reach and grasp of a cup of coffee while talking on the phone or reach and grasp goods from store shelves while recalling shopping list^8^.

Typically, a dual-task paradigm is used to investigate whether and to what extent control of a motor action requires attentional resources. Based on limited processing capacity theory, if a motor and cognitive task compete for shared attentional resources, performing the two task simultaneously may result in disruption of performance in one or both task, known as dual-task interference (for example, enhanced error and slower performance compared with the single-task condition)^9^. Individuals are frequently challenged by dual-task conditions in daily life, hence, flexible adaptation to the changing motor and cognitive requirements of daily functions, as well as environment is necessary for successfully and independently performing activities of daily livings (ADLs)^10^.

Stroke survivors may experience greater dual-task interference compared to healthy subjects because of impaired cognitive and motor function^11^. Although the effects of dual-task have been widely studied on balance and gait in stroke survivors^11,12^, few studies have been conducted on the effects of dual-task on upper limb function of these patients. In this regard, Shin et al. reported a significant dual-task effect on upper limb movement smoothness and reach error in chronic stroke survivors using a robotic-assisted planar reaching. However, they did not compare stroke survivors with healthy participants^10^. Bank et al. used a virtual goal-directed upper limb movement (i.e., controlling the movement of the virtual mouse to collect virtual targets), which performed with or without auditory stroop task in order to compare the cognitive-motor interference in patients with neurological disorders (stroke and Parkinson’s disease) with sex-and age-matched healthy controls. They did not find greater cognitive-motor interference in stroke participants than the healthy controls. They explained this finding might be related to their measure that was not precise. They suggested using a more precise measure for assessing upper limb motor control such as a motion analysis system^13^. Houwink et al. used a motion analysis system to investigate the effect of an auditory stroop task on upper limb motor control in chronic stroke survivors while drawing a circle. They found dual-task interference only in the affected upper limb of patients who had moderate upper limb paresis. However, they used an experimentally designed upper limb task, not a natural everyday task such as reach and grasp, which based on their stated limitation is susceptible to learning that is different among stroke survivors and healthy subjects^14^. It remains to be determined, however, whether dual-task would affect motor control of a well-learned/skillful upper limb movement such as reach and grasp in chronic stroke survivors.

Anxiety is the second most common psychological disorders among stroke survivors^15^, affecting up to 24% of patients^15,16^. It has been suggested that anxiety symptoms persist for up to 10 years after stroke^17^ and are associated with low functional outcomes^17^, increased dependency in ADLs^18^, and decreased quality of life^17^. Anxiety increases distraction by task-irrelevant stimuli (i.e., impaired attentional control), leading to decreased processing efficiency needed for motor planning and execution of a well-learned/skillful movement^19^. Kotani et al. recently showed that anxiety disrupted the hand’s fine motor control of expert pianists through incoordination of multi-joints movements^20^. Despite the high prevalence of anxiety in stroke survivors and its potential to affect motor control, to the best of our knowledge, no attention has been paid to the effects of anxiety on upper limb motor control of these patients. Understanding the effects of anxiety on upper limb motor control is therefore needed to develop and target interventions to address this common psychological disorder, improve upper limb motor control, and enhance the independence of stroke survivors in ADLs. Therefore, the aim of this study was to investigate whether dual-task interference would be observed in upper limb motor control of stroke survivors when performing a well-learned everyday motor task compared with age-and sex-matched healthy subjects. The study also aimed to determine the effect of anxiety on upper limb motor control of these patients.

## Results

Evaluation of demographic data (Table 1) revealed no statistically significant difference among the groups, except for their anxiety level (i.e. the score of anxiety subscale of the Hospital Anxiety and Depression Scale (HADS-A), F_(3, 64)_ = 180.08, P < 0.0001; Geriatric Anxiety Inventory (GAI) score, F_(3, 64)_ = 65.78, P < 0.0001). The results of post hoc analysis showed greater anxiety levels of the stroke and control participants with high anxiety (HA-stroke group and HA-control group, respectively) compared with both stroke and control participants with low anxiety (LA-stroke group and LA-control group, respectively). Table 2 shows the descriptive of the kinematic measures under different conditions.

**Table 1:** Demographic and clinical characteristics of the participants.

	LA-control group (n = 17)	HA-control group (n = 17)	LA-stroke group (n = 17)	HA-stroke group (n = 17)	P value
Sex (female/male), n	8/9	8/9	8/9	8/9	–
Dominant side (right/left), n	16/1	16/1	16/1	16/1	–
Affected side (right/left), n	–	–	8/9	8/9	–
Lesion type (ischemia/hemorrhage), n	–	–	15/2	13/4	0.37
**Stroke localization**					
BG (right/left), n	–	–	2/2	2/1	0.86
BG, IC (right/left), n	–	–	2/1	2/2	
CR (right/left), n	–	–	1/1	1/1	
PC (right/left), n	–	–	2/1	2/2	
FC, PC (right/left), n	–	–	1/2	1/1	
TC, PC (right/left), n	–	–	1/1	1/1	
Time since stroke (months), mean ± SD [range]	–	–	33.65 ± 22.28 [10–84]	39.65 ± 20.24 [12–75]	0.42
Age (years), mean ± SD [range]	56.59 ± 9.17 [45–66]	57.12 ± 7.74 [44–69]	58.24 ± 11.55 [44–69]	55.76 ± 9.72 [44–66]	0.90
Pain (VAS), mean ± SD [range]	0.59 ± 1.32 [0–4]	0.94 ± 1.25 [0–4]	1.82 ± 1.98 [0–5]	1.59 ± 1.83 [0–5]	0.11
MMSE (score), mean ± SD [range]	28.06 ± 1.20 [26–30]	27.76 ± 1.35 [26–30]	27.00 ± 1.87 [23–30]	26.82 ± 2.13 [23–30]	0.10
HADS-A (score), mean ± SD [range]	1.53 ± 1.66 [0–5]	12.06 ± 1.30* [11–15]	1.88 ± 2.23 [0–7]	13.00 ± 2.32* [11–18]	< 0.0001
GAI (score), mean ± SD [range]	1.53 ± 1.94 [0–5]	10.94 ± 1.39* [9–14]	3.24 ± 2.27 [0–6]	11.88 ± 3.52* [8–18]	< 0.0001
BAI (score), mean ± SD [range]	3.18 ± 2.16 [0–6]	15.88 ± 5.62* [9–29]	4.41 ± 4.89 [0–8]	16.76 ± 8.64* [10–37]	< 0.0001
Fugl–Meyer assessment, mean ± SD [range]	–	–	55.12 ± 4.71 [50–61]	58.29 ± 6.50 [50–64]	0.11
**Trail making A**					
Number of error, mean ± SD [range]	0.00 ± 0.00 [0–0]	0.18 ± 0.53 [0–2]	0.41 ± 0.79 [0–3]	0.47 ± 0.87 [0–3]	0.14
Time (s), mean ± SD [range]	47.41 ± 19.83 [29–115]	55.93 ± 37.58 [36–157]	73.11 ± 33.56 [30–145]	78.16 ± 55.82 [32–250]	0.08
**Trail making B**					
Number of error, mean ± SD [range]	1.59 ± 1.54 [0–5]	1.53 ± 1.74 [0–5]	2.13 ± 1.36 [0–4]	2.63 ± 1.09 [0–5]	0.12
Time (s), mean ± SD [range]	117.01 ± 48.50 [71–229]	118.46 ± 58.88 [71–247]	137.36 ± 63.08 [60–289]	164.24 ± 67.21 [72–290]	0.09
Forward digit span test, mean ± SD [range]	7.47 ± 0.94 [6–9]	7.06 ± 1.14 [5–9]	7.35 ± 2.57 [5–10]	6.35 ± 1.11 [5–8]	0.18
Backward digit span test, mean ± SD [range]	4.29 ± 0.77 [3–6]	4.65 ± 1.00 [3–7]	3.88 ± 1.36 [2–6]	3.76 ± 1.39 [2–6]	0.11
Reach distance, mean ± SD [range]	43.23 ± 5.97 [37–54]	43.18 ± 5.32 [38–53]	38.47 ± 6.84 [30–50]	39.46 ± 7.49 [29–50]	0.07

LA, Low Anxiety; HA, High Anxiety; BG, Basal Ganglia; IC, Internal Capsule; CR, Corona Radiate; PC, Parietal Cortex; FC, Frontal Cortex; TC, Temporal Cortex; VAS, Visual Analog Scale; MMSE, Mini-Mental State Examination; HADS-A, Anxiety subscale of Hospital Anxiety and Depression Scale; GAI, Geriatric Anxiety Inventory; BAI, Beck Anxiety Inventory.

*Shows the significant difference compared with the LA-control group.

**Table 2:** Descriptive data (mean ± SD) of reach and grasp kinematic measures in different conditions.

Kinematic measures	Condition	LA-control group (n = 17)	HA-control group (n = 17)	LA-stroke group (n = 17)	HA-stroke group (n = 17)
NMT (s/cm)	Single-task	0.031 ± 0.008	0.03 ± 0.006	0.061 ± 0.018	0.085 ± 0.033
	Easy dual-task	0.034 ± 0.008	0.034 ± 0.009	0.067 ± 0.024	0.101 ± 0.039
	Difficult dual-task	0.037 ± 0.008	0.09 ± 0.04	0.094 ± 0.023	0.122 ± 0.041
PV (cm/s)	Single-task	117.19 ± 19.44	120.20 ± 16.77	88.62 ± 38.54	65.96 ± 21.72
	Easy dual-task	112.66 ± 20.51	117.41 ± 16.40	84.004 ± 34.72	56.45 ± 22.80
	Difficult dual-task	108.27 ± 19.86	90.01 ± 7.89	75.72 ± 32.61	46.99 ± 20.12
PPV (%)	Single-task	60.18 ± 3.72	60.71 ± 3.66	39.96 ± 10.51	30.81 ± 15.42
	Easy dual-task	57.65 ± 4.29	58.09 ± 3.28	37.70 ± 11.19	26.67 ± 13.22
	Difficult dual-task	53.42 ± 5.03	44.25 ± 2.99	32.5 ± 9.08	19.14 ± 11.01
MGA (cm)	Single-task	12.9 ± 1.27	13.15 ± 0.35	11.91 ± 1.56	11.88 ± 1.06
	Easy dual-task	12.71 ± 1.3	12.96 ± 0.25	11.62 ± 1.12	10.52 ± 0.9
	Difficult dual-task	12.57 ± 1.37	12.87 ± 0.16	11.39 ± 1.67	10.21 ± 0.95
PMGA (%)	Single-task	67.23 ± 8.89	67.99 ± 3.30	75.07 ± 10.4	82.89 ± 9.67
	Easy dual-task	69.01 ± 8.21	68.01 ± 3.28	77.47 ± 9.73	87.96 ± 10.08
	Difficult dual-task	72.02 ± 9.59	74.12 ± 3.50	81.7 ± 9.99	92.74 ± 7.41

SD; standard deviation; LA, Low Anxiety; HA, High Anxiety; NMT, Normalized movement time; PV, Peak velocity; PPV, Percentage of movement time in which peak velocity occurred; MGA, Maximum grasp aperture; PMGA; Percentage of movement time in which MGA occurred.

### Reach and grasp performance/kinematic measures

**Figure 1:**
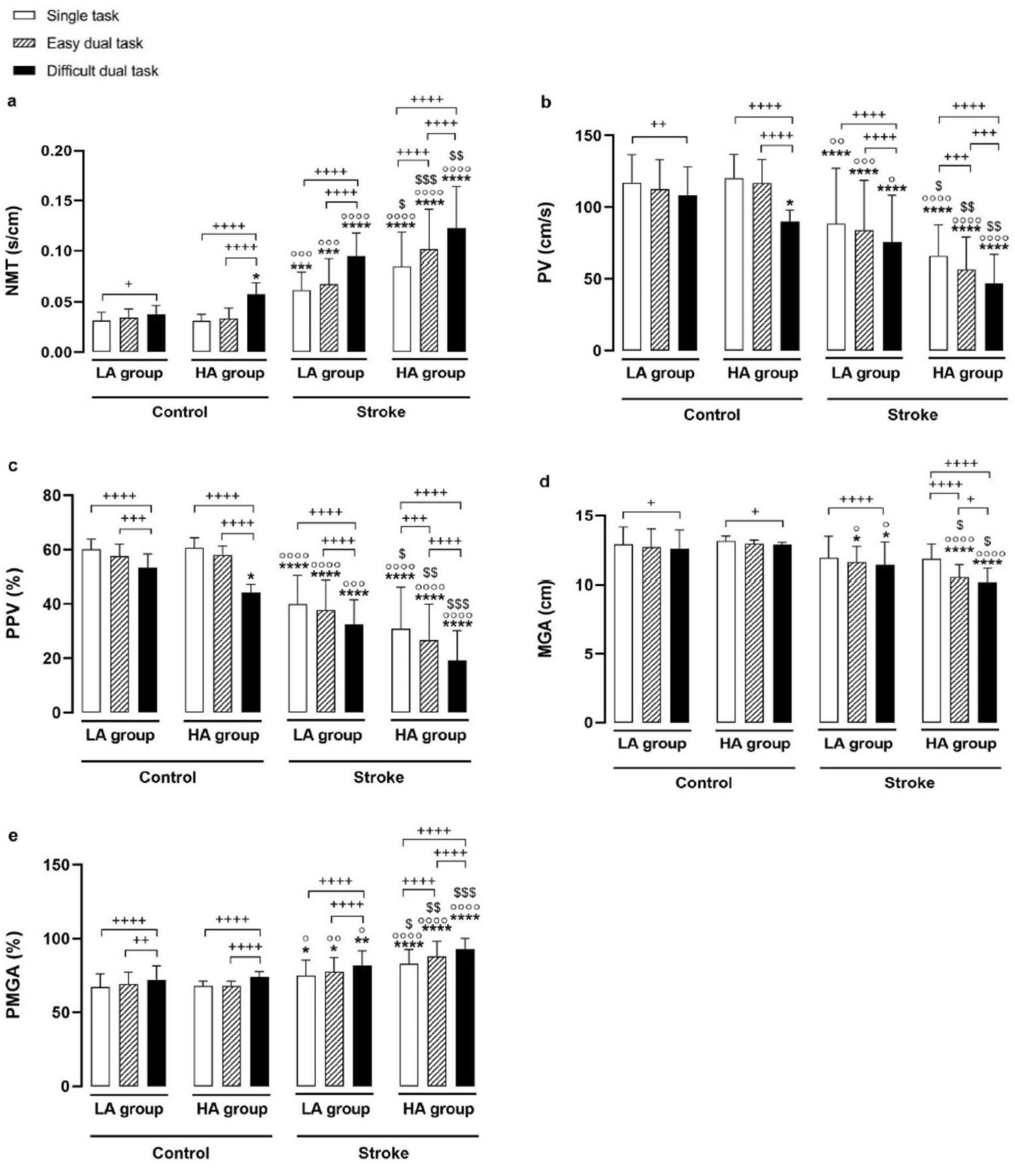
Plot of the stroke by anxiety by condition interaction on reach and grasp kinematic measures: (**a**) Normalized movement time (NMT), (**b**) Peak velocity (PV), (**c**) Percentage of movement time in which PV occurred (PPV), (**d**) Maximum grasp aperture (MGA), and (**e**) Percentage of movement time in which MGA occurred (PMGA). *P < 0.05, **P < 0.01, ***P < 0.001, and ****P < 0.0001 compared with LA-control group; °P < 0.05, °°P < 0.01, °°°P < 0.001, and °°°°P < 0.0001 compared with HA-control group; ^$^P < 0.05, ^$$^P < 0.01, and ^$$$^P < 0.001 compared with LA-stroke group; ^+^P < 0.05, ^++^P < 0.01, ^+++^P < 0.001, and ^++++^P < 0.0001.

The results yielded a significant main effect of stroke (control vs. stroke), anxiety (LA vs. HA) and condition (single-task vs. easy dual-task vs. difficult dual-task) on normalized movement time (NMT) and reach kinematic measures (i.e. peak velocity (PV) and percentage of movement time in which PV occurred (PPV)). The stroke by anxiety, stroke by condition, and anxiety by condition interactions as well as stroke by anxiety by condition interaction were also significant for NMT, PV and PPV (P ≤ 0.05) (Table 3). As illustrated in Fig. 1a–c, both LA- and HA-stroke groups showed higher NMT and lower PV and PPV than the LA- and HA-control groups in both single- and dual-task conditions. The comparison of the HA-stroke group and LA-stroke group was in a similar direction. In addition, the HA-stroke group showed less efficient motor control in both easy and difficult dual-task conditions (as indicated by higher NMT and lower PV and PPV) compared with the single-task condition and greater effect on motor control was found in the difficult dual-task condition. Conversely, in the LA-stroke, LA-control and HA-control groups, the results found less efficient motor control only in the difficult dual-task condition as compared to single-task condition.

**Table 3:** Summary of analysis of variance of kinematic measures: F ratios, P values, and effect sizes by variable.

	NMT (s/cm)			PV (cm/s)			PPV (%)			MGA (cm)			**PMGA (%)**		
	F	P	${\upeta }_{p}^{2}$	F	P	${\upeta }_{p}^{2}$	F	P	${\upeta }_{p}^{2}$	F	P	${\upeta }_{p}^{2}$	**F**	**P**	${{\varvec{\upeta}}}_{{\varvec{p}}}^{2}$
**Main effect**															
Stroke	89.87	0.000	0.58	53.26	0.000	0.45	141.34	0.000	0.69	38.46	0.000	0.38	46.75	0.000	0.42
Anxiety	10.41	0.002	0.14	6.93	0.01	0.10	11.31	0.001	0.15	1.04	0.31	0.02	7.21	0.009	0.10
Condition	243.36	0.000	0.79	108.53	0.000	0.63	229.66	0.000	0.78	72.68	0.000	0.53	109.55	0.000	0.63
**Interaction effect**															
Stroke × anxiety	4.12	0.04	0.06	4.11	0.04	0.06	4.16	0.04	0.061	3.97	0.05	0.06	5.61	0.02	0.08
Stroke × condition	29.69	0.000	0.32	4.04	0.02	0.06	3.74	0.03	0.06	22.57	0.000	0.26	5.95	0.003	0.08
Anxiety × condition	13.27	0.000	0.17	18.32	0.000	0.22	27.11	0.000	0.30	12.26	0.000	0.16	3.28	0.04	0.05
Stroke × anxiety × condition	15.75	0.000	0.20	10.25	0.000	0.14	6.89	0.001	0.10	14.89	0.000	0.19	3.27	0.04	0.05

NMT, Normalized movement time; PV, Peak velocity; PPV, Percentage of movement time in which peak velocity occurred; MGA, Maximum grasp aperture; PMGA; Percentage of movement time in which MGA occurred.

Regarding the grasp component, the results showed a significant main effect of stroke, anxiety, and condition on maximum grasp aperture (MGA) and percentage of movement time in which MGA occurred (PMGA), with the exception of main effect of anxiety for MGA. The interaction effect of stroke by anxiety, stroke by condition, and anxiety by condition was also significant for both MGA and PMGA (P ≤ 0.05). The results also showed a significant three-way interaction of stroke by anxiety by condition for both MGA (F_(2, 128)_ = 14.89, P < 0.0001) and PMGA (F_(2, 128)_ = 3.27, P = 0.04) (Table 3). The results of multiple comparisons showed that MGA was not significantly different among the four groups under the single-task condition. However, PMGA was significantly greater in LA- and HA-stroke groups compared to the LA- and HA-control groups, and in the HA-stroke group in comparison with the LA-stroke group. Performing a concurrent cognitive task led to a decrease in MGA and an increase in PMGA in all four groups, with a greater extent in the stroke groups especially the HA-stroke group. This dual-task effect was significant in both easy and difficult dual-task conditions in the HA-stroke group, while it was significant only in the difficult dual-task condition in the LA-stroke, LA-control and HA-control groups (Fig. 1d,e).

### Cognitive function

Table 4 shows the descriptive of the cognitive errors under different conditions. The results showed that cognitive error was not affected by the main effect of stroke (F_(1, 64)_ = 2.80, P = 0.10, ${\upeta }_{p}^{2}$ = 0.04), and anxiety (F_(1, 64)_ = 1.42, P = 0.24, ${\upeta }_{p}^{2}$ = 0.02), as well as the interaction effect of stroke by anxiety (F_(1, 64)_ = 0.40, P = 0.53, ${\upeta }_{p}^{2}$ = 0.006), stroke by condition (F_(1, 64)_ = 0.36, P = 0.55, ${\upeta }_{p}^{2}$ = 0.006), anxiety by condition (F_(1, 64)_ = 0.15, P = 0.70, ${\upeta }_{p}^{2}$ = 0.002), and stroke by anxiety by condition (F_(1, 64)_ = 0.009, P = 0.92, ${\upeta }_{p}^{2}$ = 0.000). However, the main effect of condition was significant for cognitive error (F_(1, 64)_ = 126.23, P < 0.0001, ${\upeta }_{p}^{2}$ = 0.66), indicating a greater cognitive error in the difficult dual-task condition than the easy dual-task condition.

**Table 4:** Descriptive data (mean ± SD) for cognitive error in different conditions.

Cognitive error	LA-control group (n = 17)	HA-control group (n = 17)	LA-stroke group (n = 17)	HA-stroke group (n = 17)
Easy dual-task	0.038 ± 0.11	0.058 ± 0.17	0.098 ± 0.28	0.31 ± 0.53
Difficult dual-task	1.25 ± 0.61	1.39 ± 0.89	1.48 ± 1.21	1.76 ± 1.21

SD; standard deviation, LA; Low Anxiety, HA; High Anxiety.

## Discussion

The current study aimed to determine the effects of dual-task on spatiotemporal control of reach and grasp in chronic stroke survivors compared with age-and sex-matched healthy subjects and to investigate the effects of anxiety on reach and grasp motor control in these patients. To the best of our knowledge, this is the first study that has investigated the effect of dual-task and anxiety on an everyday functional upper limb movement in chronic stroke survivors. A major finding of this study was that stroke survivors especially those with a high level of anxiety, showed a greater decrement in dual-task conditions than the single-task condition on kinematic measures of reach and grasp when compared with age- and sex-matched healthy subjects. Interestingly, although the high-demanding dual-task condition revealed significant dual-task interference on reach and grasp kinematic measures in all four groups (i.e. LA-stroke, HA-stroke, LA-control, and HA-control groups), the low-demanding dual-task condition led to the significant decrement of reach and grasp performance only in the HA-stroke group.

In agreement with previous studies, our findings showed impaired motor control of reach and grasp in both LA- and HA-stroke groups compared with age- and sex-matched controls (both LA- and HA-control groups) including greater movement time^4,5,21^, lower PV^3,4,22^ and lower PPV^2,3^. Longer NMT in stroke survivors may be caused by their lower PV, which in turn may be the result of poor strength in these patients^23^. Lower PPV in stroke groups indicates longer deceleration time, which provides greater time to on-line adjust movement using proprioceptive and visual feedback mechanisms to compensate for stroke-induced impairments of neural networks and motor command generation^24^.

The results of this study showed that MGA was significantly smaller in the LA- and HA-stroke groups compared with both LA- and HA-control groups under dual-task conditions. However, the difference between the groups was not significant under the single-task condition. Previous studies have investigated reach and grasp kinematics in the stroke survivors only under the single-task conditions and reported conflicting results about the MGA. In agreement with the results of the current study, Harvey et al.^25^ and Baak et al.^26^ did not find a significant difference between stroke survivors and healthy subjects regarding their MGA in a reach and grasp task. In contrast, Nowak et al.^4^ reported greater MGA in stroke survivors. This discrepancy can be explained by the task constraints exerted in Nowak et al. study, in which subjects had to grasp a disk (3 cm width and 9 cm diameter) only with the thumb and index fingers, while the thumb was in opposition to all fingers in the present study.

Further, in line with the previous studies^3,26^, the result of this study showed delayed MGA (i.e. greater PMGA) in stroke groups as compared with the control groups, indicating coordination deficits in the coupling between the transport and grasp component of reach and grasp task in these patients. Greater PMGA in stroke survivors has been attributed to their difficulty in generating force in finger extensors to open their hands, which may result in more time required for achieving MGA^3^. Another important finding of this study was that dual-task affected the kinematic measures of both transport and grasp components (i.e. increased NMT, decreased PV, PPV, and MGA as well as increased PMGA) of the reach and grasp task in the four groups. However, the effects of dual-task on reach and grasp kinematic measures were significantly increased by increasing the difficulty level of concurrent cognitive task and were significantly greater in stroke survivors, especially the HA-stroke group compared with the LA- and HA-control groups. This finding supports recent studies that found performing a well-learned/skillful movement such as reach and grasp requires attentional resources and is not fully automated, even in healthy subjects. In this regard, Hesse et al.^7^ also found a significant decrease in MGA and an increase in PMGA in young healthy subjects under dual-task conditions compared with the single-task condition. Because the cognitive task used in the current study (backward digits string) has little overlap with the motor task (reach and grasp as a visually-guided goal-directed motor action), it is reasonable to suggest that dual-task interference may occur due to shared working memory-related attention. More specifically, motor planning of reach and grasp requires the visual and spatial representation of the target object, which in turn needs to attentional resources. Thus, it may be suggested that backward digits string task and reach and grasp task may compete for shared finite attentional resources, leading to dual-task interference in reach and grasp kinematics in the four groups with a greater extent in chronic stroke survivors, especially those with the high level of anxiety. A possible explanation for greater dual-task effect on reach and grasp kinematic measures in stroke survivors may increase attentional demands of controlling movement due to stroke-induced sensory and motor impairments^11^ and the tendency of stroke survivors to consciously control their movements^27^ which may overload the limited attentional resources and lead to insufficient attentional resources for performing reach and grasp under dual-task condition.

The most interesting finding of this study was the worse performance of the HA-stroke group on the reach and grasp kinematic measures compared with the LA-stroke group under both single- and dual-task conditions. However, the worse performance of the HA-control group on the reach and grasp kinematic measures compared with the LA-control group was only observed in the difficult dual-task condition. Anxiety leads to depletion of attentional resources by allocating them to task-unrelated stimuli^28^. It has been suggested that this anxiety-induced distraction decreases processing efficiency, leading to impairments in goal-directed action in both motor and cognitive aspects^29^. Also, anxiety leads to a cognitive effort to apply conscious control on otherwise more automatically controlled movement^30^. Allocating much attention to consciously control a well-learned motor task, such as reach and grasp may result in step-by-step controlling of the movement, leading to decreased performance^31^. Therefore, a high level of anxiety may intensify the increased attentional demands of motor control and tendency to consciously control movements in chronic stroke survivors, leading to overload of limited attentional resources, and ultimately worse insufficiency of reach and grasp motor control.

Regarding the cognitive performance under the dual-task condition, although the cognitive errors were increased from the healthy control groups to the LA-stroke group and to the HA-stroke group, the difference between the four groups did not reach a significant level. One possible explanation for this result may be that the attentional demands of the digits string test used in the current study may have not been sufficient to show the effects of anxiety on the cognitive function of stroke survivors under the dual-task conditions. Future research should consider different cognitive tasks with greater attentional demands to better understand the effect of anxiety on the cognitive function of stroke survivors under dual-task conditions. The results also found that cognitive error significantly increased by increasing the cognitive task difficulty in the four groups. This might be explained by the limitation of the brain’s processing capacity. Besides, by increasing the difficulty of concurrent cognitive task, subjects may prioritize their reach and grasp motor function over cognitive performance, leading to higher cognitive error under difficult dual-task conditions.

The results of this study bring forth some potential clinical implications. Foremost, it is important to recognize that anxiety may aggravate stroke-induced impairments of reach and grasp motor control. Hence, considering the assessment of anxiety and designing appropriate pharmacological/behavioral interventions is important to improve upper limb motor control in chronic stroke survivors. Second, although clinical tests of upper limb (e.g. Fugl-Meyer Assessment, Wolf motor function test, etc.) provide useful information to clinicians, they do not consider the function under the dual-task condition and thus, may result in underestimation of impairments in commonly occurred dual-task conditions in real daily activities. This may explain the discrepancy usually observed between the upper limb capacity of stroke survivors (i.e. the ability to perform tasks by the affected limb) and actual use of the affected upper limb in everyday activities^32^. Therefore, assessment of upper limb motor function and motor control strategies under challenging dual-task conditions along with common clinical measures may provide a more accurate estimation of upper limb function in chronic stroke survivors while performing daily activities. Moreover, designing dual-task rehabilitation interventions for upper limb function may be useful for chronic stroke survivors, and needs to be investigated in future studies.

Finally, some limitations of the present study should be acknowledged. First, all participants in both HA- and LA-stroke groups had the Fugl-Meyer score ≥ 50, which shows mild upper limb motor impairment^33,34^. Thus, the results of the current study could not be generalized to stroke survivors with moderate/severe upper limb motor impairment. Second, we used a harness to avoid trunk compensatory movements in this study, which are common among stroke survivors. One may assume that restraining the trunk may lead to increased attentional demand in the HA-stroke group. However, there was not a significant difference between the LA- and HA-stroke groups regarding their upper limb motor impairment based on the Fugl-Meyer score. Further, it has been shown that stroke survivors with moderate to severe upper limb motor impairment recruited trunk movements to compensate for motor deficits, while stroke survivors with mild upper limb motor impairment tend to use movement patterns similar to healthy subjects rather than trunk compensatory movements^35,36^. However, investigating the effects of dual-task on trunk movements while performing an upper limb motor task would shed light on the use of compensatory strategies under dual-task conditions. Besides, combining neurophysiological measures (e.g. electromyography, electroencephalography and functional magnetic resonance imaging (fMRI) with kinematic measures of upper limb motor control can provide useful information about motor control-related neural processes of upper limb function in stroke survivors.

In conclusion, the findings of this study indicated greater inefficiency of reach and grasp motor control in chronic stroke survivors compared with age- and sex-matched healthy subjects as evidenced by longer movement time, lower and earlier peak velocity, as well as delayed and smaller hand opening. This study also provides for the first time evidence about the effect of anxiety on spatiotemporal control of reach and grasp in these patients. More specifically, the current study reveals that HA-stroke survivors have worse motor control of reach and grasp compared with the LA-stroke survivors under both single- and dual-task conditions. Thus, due to the importance of reach and grasp in object manipulation, HA-stroke survivors may have less capacity to cope with the increased demand for object manipulation in dual-task conditions, which are frequently seen in everyday life.

## Methods

### Participants

**Figure 2:**
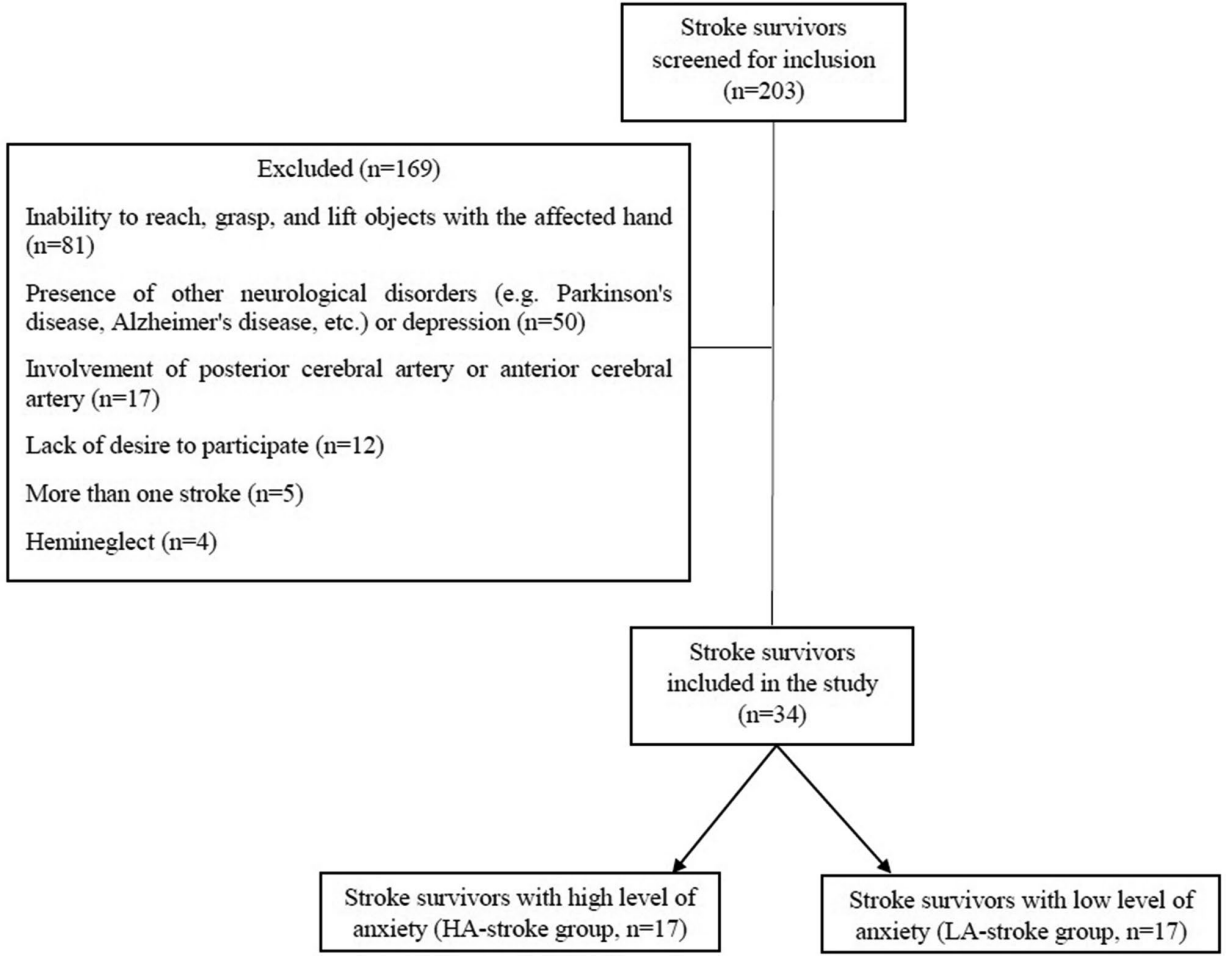
Flowchart of the inclusion of stroke participants. LA- and HA-stroke survivors were matched based on the side of their affected hand, age, and sex. In addition to LA- and HA-stroke participants, 34 age-, and sex-matched healthy subjects also participated in the study.

Thirty-four stroke survivors were recruited from the neurologic disorders clinics and rehabilitation centers of the university and were grouped according to HADS-A^37^ and Geriatric GAI^38^ scores to LA-stroke (score < 11 on the HADS-A and score < 7 on the GAI; n = 17) and HA-stroke (score ≥ 11 on the HADS-A and score ≥ 7 on the GAI; n = 17) (Fig. 2). The LA- and HA-stroke groups were matched in terms of the side of the affected hand, age, and sex. Inclusion criteria for stroke participants were: (1) diagnosis a single, unilateral stroke within the territory of the middle cerebral artery at least 6 months earlier verified by brain imaging; (2) ability to reach, grasp and lift objects with the affected hand; and (3) score ≥ 23 on the Mini-Mental State Examination (MMSE)^39^. Exclusion criteria were: (1) the presence of other neurological disorders or depression (i.e., score > 7 on the depression subscale of HADS); (2) the presence of hemineglect (i.e., score ≥ 44 on the star cancellation test)^40^; (3) any comorbidity affecting upper limb function; and (4) the presence of fatigue (i.e., score ≥ 36 on the Fatigue Severity Scale (FSS)^41^). The HA-stroke group did not have different CNS active medication (to treat anxiety) than the LA-stroke group. Thirty four age- and sex-matched healthy subjects without any neurological or musculoskeletal disorders affecting upper limb function, depression (i.e. score ≤ 7 on the HADS-D) and fatigue (i.e. score < 36 on the FSS) participated as controls. Healthy participants also were grouped to low anxiety-control (LA-control; n = 17) and high anxiety-control (HA-control; n = 17) according to the score of the HADS-A^37^ and GAI^42^. All subjects in the four groups had a normal or corrected-to-normal vision and were evaluated by the Trail Making Test (part A and B) and forward and backward digit span test to assess cognitive function. The pain was also assessed in all participants using the Visual Analog Scale (VAS). The experimental procedures were approved by the Ethics Committee of the Iran University of Medical Sciences (IR.IUMS.REC.1396.9221525203) and all methods were carried out in accordance with the Helsinki Declaration. All participants gave written informed consent to participate in the study.

### Experimental procedures/tasks

**Figure 3:**
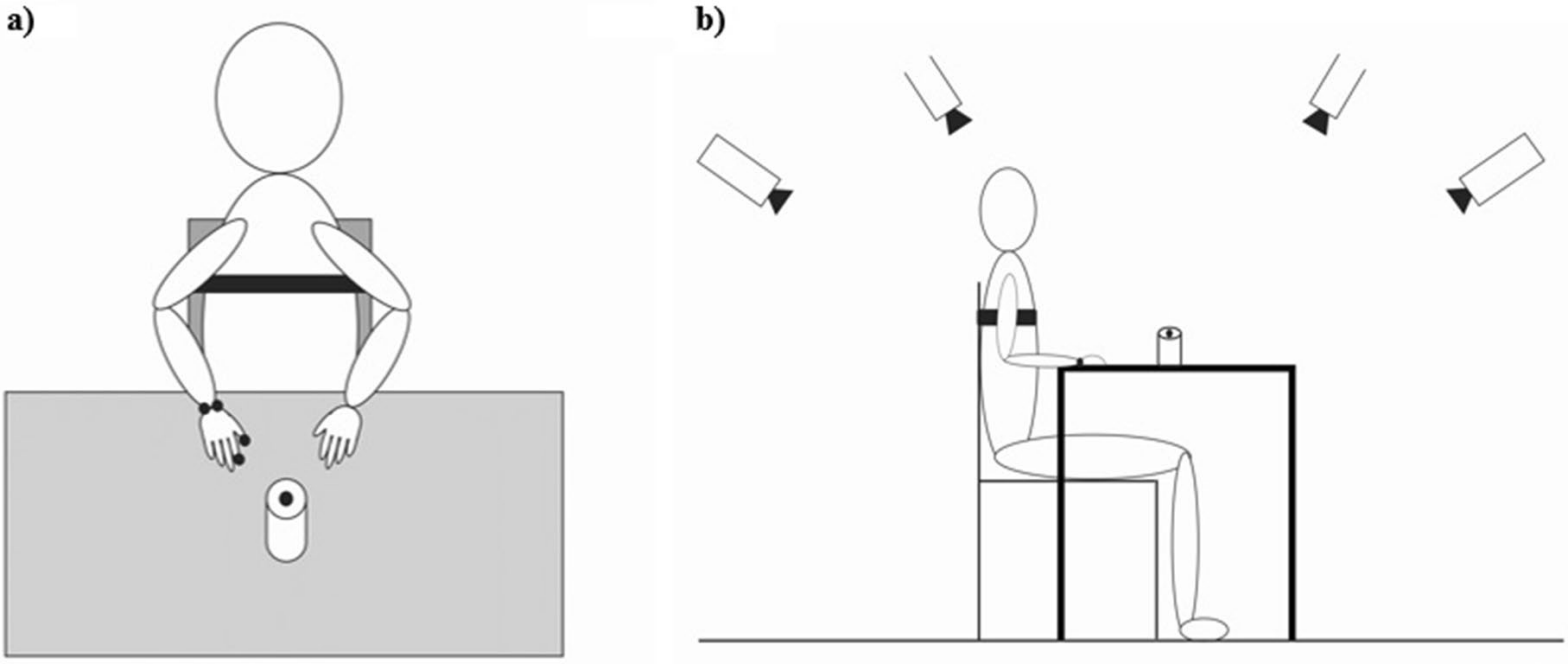
Representation of a participant seated on the chair, in front of a table (anterior view (**a**) and lateral view (**b**)) where the target object (a jar with 8 cm diameter and 12 cm height) is placed. Reflective markers were placed on the jar and the styloid process of ulna and radius as well as nails of the index finger and thumb of the reaching upper limb.

We tested reach and grasp movement to a target (a jar with 8 cm and 12 cm in diameter and height respectively) while participants sat in a straight-backed chair in front of a table and their trunk strapped to the back of the chair in order to minimize compensatory movements of the trunk, which are common among stroke survivors. Stroke participants performed the task with their affected upper limb. Control subjects were matched with stroke survivors in terms of using their dominant or non-dominant hand to perform the task. At the beginning of the experiment, the participant’s reaching hand was positioned with his/her hand resting on the table in a pronated position, shoulder in the neutral position, and elbow flexed between 70 and 90°. The target object was positioned at a maximum functional reaching distance determined by the maximum reachable distance for each participant along with his/her body midline (Fig. 3). Participants were asked to reach toward, grasp, and lift the target using their thumb and the rest of the fingers when they heard the word “start”. They were asked to do this task at their self-preferred speed. The task was performed under three experimental conditions, which were presented in random order: single-task condition (reach and grasp the jar without performing a simultaneous cognitive task), as well as easy and difficult dual-task condition (reach and grasp the jar while simultaneously performing the easy and difficult cognitive task (i.e. backward digit task)). The participants were instructed to do both cognitive and motor tasks to their best ability under dual-task conditions. The number of maximum digit span of each participant plus one was set as a difficult cognitive task. The number of digits presented in the easy cognitive task was half of the difficult cognitive task, which rounded up if the number was odd. Three trials of each condition were conducted and participants were asked to close their eyes between trials. In dual-task conditions, participants were needed to hear a sequence of digits before starting the trial, rehearse the sequence mentally in reverse order during the trial, and report the sequence in reverse order to the examiner at the end of the trial. The difficulty of the cognitive task was set individually based on the maximum digit span of each participant as previously described^43^. Three types of cognitive errors were recorded including incorrect number, order error, or omission error.

The kinematic data were recorded using a 6-camera motion analysis system (VICON, 2.5) at 120 Hz. Reflective markers were placed on the jar and the following points of the reaching upper limb: the styloid process of ulna and radius, nails of the index finger and thumb.

### Data analysis

The kinematic recording was analyzed through custom-written Matlab code, where they first smoothed using a second-order, 10 Hz low-pass Butterworth filter. Based on Jeannerod's model, reach and grasp movement includes two components: (1) Transport or reaching, during which information about the spatial location of the target object is extracted and used to bring the hand towards the object; and (2) Grasp or manipulation, during which information about the intrinsic features of the object (e.g. shape and size) are extracted, which determined an appropriate grasp pattern^44^. Both reach and grasp components occur simultaneously and are firmly coordinated and temporally coupled during movement execution^45^. The following kinematic measures were calculated for each trial. Movement time, as a global measure of reach and grasp motor control, was defined as the time between the onset of the movement and the end of the action (i.e. lifting the jar). Since the reaching distance varied among participants, movement time was normalized (NMT) by dividing it by reaching distance for each participant to correct for differences in reaching distance. For the transport/reach component, the amount of PV and PPV were calculated. There is a close relationship between movement time and PV. It has been suggested that velocity profiles indicate the effectiveness of motor control^46^. For the grasp/manipulation component, MGA (the maximum three-dimensional distance between the index finger and thumb markers) and PMGA were calculated. To configure and orient the hand for a successful grasp, visual system should first process various visual cues regarding the intrinsic properties of the target object, then integrates these cues into an estimate of the shape and size of the target object, which is then transformed into proper motor programs (i.e. visuomotor transformations)^47–49^. MGA is considered as an index of visuomotor transformations that occurs during grasp^50^. PMGA is a measure of temporal coupling between reach and grasp components^51^.

### Statistical analysis

The normality assumption of the data was satisfied based on Shapiro–Wilk test results. To assess the comparability of the general characteristics among the groups, one-way analysis of variance (ANOVA) or independent sample t-test was used for continuous variables and Chi-square test was used for categorical variables. For kinematic parameters and cognitive error, a mean value of three trials was used for statistical analysis. To investigate the effect of stroke, anxiety and dual-task on different kinematic parameters, a three-way mixed-model ANOVA was used, with a stroke (control vs. stroke) and anxiety (low anxiety vs. high anxiety) as between-group factors and condition (single-task vs. easy dual-task vs. difficult dual-task) as a within-group factor. The effects of stroke, anxiety and dual-task on the cognitive function were evaluated using the same model, with a stroke (control vs. stroke) and anxiety (low anxiety vs. high anxiety) as between-group factors and condition (easy dual-task vs. difficult dual-task) as a within-group factor.
